# Early life programming of health and disease: The long‐term consequences of obesity in pregnancy

**DOI:** 10.1111/jhn.13023

**Published:** 2022-05-10

**Authors:** Simon C. Langley‐Evans

**Affiliations:** ^1^ School of Biosciences, University of Nottingham Sutton Bonington Campus Loughborough UK

**Keywords:** cardiovascular disease, diabetes, disease, life phase, obesity, pregnancy, therapeutic areas

## Abstract

The prevalence of overweight and obesity is rising in all parts of the world and, among young women, it presents a very clear danger during pregnancy. Women who are overweight or who gain excessive weight during pregnancy are at greater risk of complications in pregnancy and labour, and are more likely to lose their child to stillbirth or die themselves during pregnancy. This narrative review considers the evidence that, in addition to increasing risk of poor pregnancy outcomes, obesity has the capacity to programme foetuses to be at greater risk of cardiometabolic disorders later in life. An extensive body of evidence from prospective and retrospective cohorts, as well as record linkage studies, demonstrates associations of maternal obesity and/or gestational diabetes with cardiovascular disease, as well as type 1 and type 2 diabetes. Studies in animals suggest that these associations are underpinned by adaptations that occur in foetal life, which remodel the structures of major organs, including the brain, kidney and pancreas.

## Highlights

- Foetal exposure to maternal obesity is associated with greater risk of childhood obesity, cardiovascular disease, type 1 and type 2 diabetes, and renal disease.
- Animal studies suggest that the programming of health and disease by maternal obesity is likely to be underpinned by remodelling of organ structures during development.
- Placental adaptations to maternal obesity, including inflammatory responses, may mediate the foetal tissue remodelling that occurs in response to maternal obesity or excessive antenatal weight gain.
- Transgenerational cycles of obesity and related disorders, with transmission through the maternal environment during pregnancy, make intervention a high public health priority. In addition to the periconceptual period, there are windows of opportunity around breastfeeding and the timely and appropriate introduction of complementary foods in infancy.

## Graphical abstract

The risk of disease at any stage of life is determined by the outcome of gene–environment interactions at all preceding life stages. The environment encountered in foetal life is particularly important in the programming of tissue structure and function. Experience of maternal obesity in foetal life is associated with a greater risk of cardiometabolic disorders and may establish a transgenerational cycle of obesity.

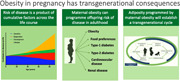

## THE LONG‐TERM CONSEQUENCES OF OBESITY IN PREGNANCY

### The determinants of health and disease

The last 30–40 years have seen a profound change in our understanding of relationships between lifestyle and chronic disease. Recognition that lifestyle factors could modulate risk of non‐communicable disease arose from the seminal works by Doll and Peto^1^ on tobacco smoking and cancer and the elucidation of relationships between dietary fat, cholesterol concentrations and the contribution of low‐density lipoprotein‐cholesterol to atherosclerosis.^2^, ^3^ Following on from this recognition came the now well‐established view that risk is heavily determined by the interaction of lifestyle factors and the genotype. The phrase ‘genes load the gun but the environment pulls the trigger’ is widely quoted,^4^ after having been coined by Judith Stern, a nutritionist from the University of California. The research that is described in the present review adds another layer to our understanding of the lifestyle–disease relationship and, as will be discussed, it is perhaps more accurate to say that genes load the gun, early life factors take aim and the environment pulls the trigger.

**Figure 1:**
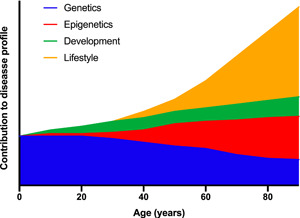
The underlying determinants of health and disease are complex and vary across the lifespan. At all stages of life, health status is a product of gene–environment interactions. In early life, genetics plays a more important role than in later life. Risk of disease at all stages of life is a product of the outcomes of gene–environment interactions at earlier stages

Figure 1 shows how different elements of broad human biology establish risk of disease at any stage of life. The contribution of genetics is strongest in the earlier years and a high proportion of non‐communicable disease in infants and children will have a strong genetic component. The influence of lifestyle (smoking, diet, alcohol, socioeconomic status, occupational exposures and behaviours) becomes stronger as we age, but will always be modified by the underlying genotype. However, the phenotype also plays a critical role. ‘Genotype’ describes the information encoded by DNA, whereas ‘phenotype’ refers to the traits the individual has when the genotype is expressed. The translation of the genotype to phenotype will be modified by epigenetic factors (tags on DNA and histone proteins that modulate gene expression in response to a range of factors, including the environment) and the environment encountered during foetal and infant development. It is helpful to regard health and disease status at any stage of life as being the product of cumulative gene–environment interactions at all previous stages of life.^5^ The outcomes of such interactions at one stage of life will establish the phenotypic traits that determine how future interactions progress. This could be regarded as a lifecourse approach to understanding disease, or to stretch Stern's gun analogy further, we might say that the aiming of the weapon prior to the environmental trigger that causes detriment becomes more focused with ageing.

**Figure 2:**
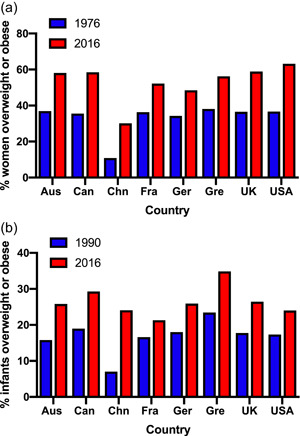
The rising prevalence of obesity. (a) Overweight and obesity among adult women in selected countries. (b) Overweight and obesity among children aged 2–4 years in selected countries. Data from Ritchie and Roser.^7^ Aus, Australia; Can, Canada; Chn, China; Fra, France; Ger, Germany; Gre, Greece; UK, United Kingdom; USA, United States of America

Because early development, particularly during the phase of life when organogenesis irreversibly establishes tissue structures, is a contributing factor to disease risk many decades later, exploring the relationship between maternal nutritional status in pregnancy and infant development is of great interest. Although there is an extensive body of evidence considering how maternal undernutrition can ‘programme’ later disease,^6^ the major nutritional concern in contemporary society relates to overweight and obesity. Figure 2 shows recent trends in overweight and obesity for adult women and infants aged 2–4 years. The marked rise in prevalence in both groups is striking, and the impact of widespread overweight among women of childbearing age upon the health of future generations is yet to be fully evaluated. The aim of this review is to discuss the evidence that links early life events to disease in later life and consider observations that indicate that maternal obesity is a key driver of non‐communicable disease in the next generation. In the review the focus will be primarily on epidemiological associations between early life and later disease, although findings from experimental animal studies will be described as a means of illustrating the mechanisms the likely mechanistic links.

## MATERNAL OBESITY AND PREGNANCY OUTCOMES

The risks associated with obesity in pregnancy manifest during the pregnancy itself, although this review focuses on the later impacts on the health of individuals who experienced maternal obesity during foetal development. Maternal weight status is an important determinant of pregnancy outcomes for both mothers and babies, and also of obstetric complications.^8^ Overweight, obesity and excessive gestational weight gain are all risk factors for poor outcomes. Optimal gestational weight gain is dependent on weight status going into pregnancy. Although women of ideal weight prepregnancy may gain up to 16 kg across pregnancy, for those who are obese, a gain over 9 kg would be considered excessive.^9^

Obesity and excessive weight gain increase the risk of miscarriage^10^ and stillbirth.^11^ Obesity also increases risk of maternal death by up to two‐fold, relative to ideal weight women, depending on the severity of the obesity.^12^ Overweight, obesity and excessive weight gain increase risk of all obstetric complications ranging from relatively minor gastrointestinal disturbances^13^ through to the more severe hypertensive disorders^14^ and gestational diabetes (GDM)^15^. It is estimated that obesity increases the risk of pre‐eclampsia by between 2‐ and 2.5‐fold^16^ and, because the clinical response to this condition is to deliver the baby early, obesity becomes a key risk factor for preterm delivery. The association between obesity and gestational diabetes increases risk of macrosomia and injury to infants during delivery.^17^ Obesity and excessive gestational weight gain also increase the risk of complications in labour. Spontaneous initiation of sustained labour is impaired, making induction more common in women with a body mass index (BMI) > 30 kg m^–2^.^18^ Interventions, including instrumented delivery and caesarean section, are more frequent in pregnancies complicated by maternal obesity.^19^, ^20^

## THE EARLY LIFE ORIGINS OF HEALTH AND DISEASE

The starting point for this review was the idea that lifelong risk of non‐communicable disease is a product of interactions between genetic and epigenetic factors with the environment at all stages of life. Disease risk at any given point in life is partly determined by prior (epi)genetic–environment interactions. The impact of such interactions during foetal development is particularly significant and exposures to adverse environments during this phase of life are said to programme later disease risk. In this context ‘programming’ refers to permanent, irreversible adaptations to the environment, which compromise capacity to maintain normal metabolic and physiological function with ageing.^6^

**Figure 3:**
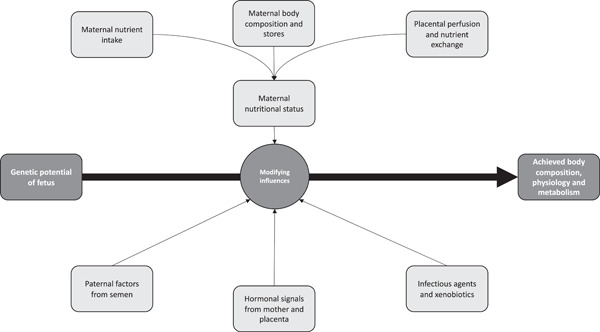
Maternal and paternal factors modify genetically determined developmental potential to determine the foetal genotype at birth

The embryos of all animal species begin development with the potential to develop and grow at a rate and to a form, that is determined by the genotype inherited from both parents. The expression of that genetic potential will lead to an achieved phenotype (Figure 3), which includes all aspects of anatomy, physiology, metabolism and endocrine functions, and hence the balance between health and disease. The early life programming concept is based on the contribution of modifying factors that alter the expression of the genotype and hence the achieved phenotype. Modifying influences on development will change the achieved phenotype at the level of individual organs, systems, tissues and even specific cell types by altering rates of cell division and differentiation. These changes will determine the number of cells and types of cells within a tissue and hence its resilience and homeostatic responses to physiological and metabolic challenges from the environment (dietary surplus or deficit, infection, trauma).^6^ Ultimately, the establishment of the phenotype from the genotype is never complete and it continues to change throughout life, although tissues are at their most plastic during the phases of organogenesis and maturation which, in humans, occur before birth. In this way, exposure to environmental factors establishes the functional lifespan of each organ, meaning the length of time over which it can maintain normal function and the capacity to withstand further adverse conditions. Beyond this functional lifespan, each tissue will decline in function and disease will develop.

The range of factors known to have a programming effect on the developing foetus is known to be broad (Figure 3). Inevitably, most factors are maternally derived as the intrauterine environment is where the foetal genotype encounters stimuli from the outside world, but there is an emerging body of evidence that suggests paternal factors carried by semen may also have programming potential. Most attention has focused on the influence of maternal nutritional status, and in particular undernutrition. The way in which that is signalled to the embryo and foetus is complex and nutritional status is, in itself, a product of maternal intakes, nutrient demands and stores.

The first compelling evidence that early life events could programme disease in adulthood was derived from ecological and retrospective cohort studies. Comparing the geographical distributions of place of birth and cause of death among more than 2 million individuals who died in England and Wales suggested a strong influence of the former upon risk of coronary heart disease in the 1970s.^21^ Similarly, the distribution of death from coronary heart disease mapped closed onto the distribution of infant death in the 1920s.^22^ This suggested that deprivation in early life was related to subsequent disease and cause of death and that this relationship persisted even when people moved to more affluent parts of the country. Further studies found strong associations between anthropometry at birth and risk of disease in adult life. Across cohorts in many countries, including the UK, Sweden, USA and India, it was noted that lower weight at birth (but still within the normal range) was associated with higher risk of blood pressure in adulthood, type 2 diabetes, insulin resistance and death from coronary heart disease.^23^, ^24^, ^25^, ^26^, ^27^, ^28^ These observations were particularly robust for type 2 diabetes and meta‐analysis suggested a 25% greater risk of adult diabetes with every 1 kg lower weight at birth.^29^

Other indices of infant anthropometry at birth were also found to be associated with risk of ill‐health in childhood and adult life. A larger head circumference, for example, was associated with greater risk of atopic wheezing in primary school age children.^30^ Thinness at birth, measured as the ponderal index (weight/length^3^), was found to associate with risk of type 2 diabetes as an adult,^31^ and a smaller abdominal circumference was associated with cardiovascular disease.^32^ Collectively, these observations led to the theory that factors that constitute an adverse environment for foetal development, result in irreversible changes to how organs and tissues develop, effectively programming their lifelong function and risk of non‐communicable disease for the exposed individual. The extremes of anthropometric indices at birth are the immediate indicator that the maternal environment has constrained genetic potential for growth. In keeping with the idea that risk of disease at any stage of life is the product of cumulative exposures to adverse factors at earlier stages, more complex analyses of retrospective cohorts showed interactions between foetal and adult factors. Risk of insulin resistance in 50‐year‐old men and women was greatest in those who were born thin (low ponderal index) but had higher body mass index as adults.^33^ Finnish women born in the 1920s and 1930s were more likely to develop coronary heart disease if they were of low birthweight and gained weight more rapidly up to the age of 9 years.^34^ Similarly, the interaction of early life factors with the genotype is evident from observational data. Associations between common single nucleotide polymorphisms (gene variants) and disease were only manifested in individuals of lower birthweight in a cohort of Finnish adults.^35^, ^36^

The originators of the programming hypothesis postulated that the principle driver of early life programming was maternal undernutrition because evidence suggested that birth anthropometry was determined by maternal nutritional status and because, at the time the participants in the retrospective cohorts were conceived and born (early 20th Century), undernutrition was considerably more common than overnutrition, overweight and obesity. Reinforcement of the nutritional programming hypothesis came from follow‐up studies of individuals conceived or born during the Dutch Famine of 1944–1945. At the end of World War II, Nazi blockade of food supplies to western Holland resulted in 6 months of famine conditions. Adults who were conceived at this time were more likely to develop obesity, type 2 diabetes and coronary heart disease than those born just before or just after the famine.^37^, ^38^ Undernutrition at different stages of foetal development had differential effects on disease in adult life. Exposure to famine in early gestation was associated with greater risk of coronary heart disease, schizophrenia and depression, whereas exposure at any stage of gestation was associated with type 2 diabetes.^37^ Similarly, foetal exposure to the Great Chinese Famine of the 1950s was associated with greater risk of ischemic heart disease and stroke, non‐alcoholic fatty liver disease and type 2 diabetes^39^, ^40^, ^41^ but not left atrial enlargement.^42^

**Figure 4:**
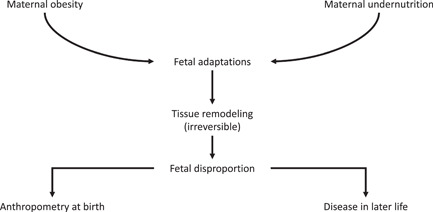
Programming of disease in later life can be driven by both maternal under‐ and overnutrition

There are different ways of viewing the relationship between anthropometry at birth and disease in adult life. Nobody would view being born small as being a direct cause of non‐communicable disease. Lower weight or thinness at birth are merely indicators of risk. There are three main schools of thought about what the observed relationship means. The simplest view is that the association represents a trade‐off in foetal life. Adverse conditions in pregnancy as a result of undernutrition or other maternal stressors either result in death of the embryo or foetus, or the conceptus survives through adaptations of tissue structures.^6^ These adaptations become permanent as they occur during organogenesis, and are subsequently disadvantageous to the adult (Figure 4). Others have considered the persistence of what appears to be maladaptation through evolution and have proposed that, when the foetus adapts to the prevailing environment encountered by its mother, it develops characteristics preparing it for the continuation of that environment after birth. Disease risk will only develop if conditions improve.^43^, ^44^ For example, conditions of undernutrition would be better responded to if an individual were programmed in foetal life to be more energetically efficient. However, if in future the individual lives in an environment where nutrition is plentiful, then they would be more likely to become obese. The third viewpoint is that the birth anthropometry–later disease association is an indication that there are genetic variants that mediate both foetal growth restriction and non‐communicable disease.^45^ For example, Warrington *et al*.^46^ reported on genome wide association analysis of maternal and offspring genotypes, birthweight and cardiometabolic disease in a population of more than 200,000 individuals. The analysis found 190 independent association signals indicating that the foetal genotype determined both birthweight and adult blood pressure. This would discount any involvement of maternal nutritional status or other putative programming factors.

The foetal programming hypothesis is also open to criticism because so much of the supporting evidence is dependent upon retrospective analysis of data gathered for other purposes. Studies that attempt to link indicators of the foetal environment with outcomes that manifest more than five decades later are inevitably vulnerable to confounding factors that cannot be fully controlled for in the statistical analysis.^6^, ^47^ In most studies, except those that have involved follow‐up of the wartime and other famines, there is no direct measure of maternal nutritional status and much is inferred from birth anthropometry as an imperfect proxy of undernutrition. A number of prospective cohort studies^48^, ^49^, ^50^ have been established to investigate the maternal nutrition relationship with offspring health, but all of these are still many decades away from yielding useful observations that can adequately confirm or refute the hypothesis.

Given the counter‐arguments to the nutritional programming hypothesis, it has been important to assess the biological plausibility of the concept using experimental animal models. These have been able to demonstrate direct programming responses to manipulation of maternal diet, in the absence of genetic variation. Tests of the maternal diet–disease relationship have shown that the concept holds true, with a high degree of confidence. Programming through maternal undernutrition has been shown in a wide range of mammalian species including pigs, sheep, guinea pigs, mice and rats.^47^ In genetically in‐bred rodents, restriction of maternal food intake, induction of iron deficiency and feeding a low protein diet in pregnancy impair foetal growth, disrupt placentation, lead to defects of glucose homeostasis, increase blood pressure and impair renal function in adult offspring, whose lifespans are reduced.^51^ Studies of non‐human primates also demonstrate that maternal undernutrition in pregnancy adversely programmes physiological and metabolic function in the exposed offspring.^52^ Critically, the animal studies have shown that maternal undernutrition can programme later disease without any effect on birthweight. This undermines the argument that epidemiological evidence of programming is explained by genetic variants that influence both foetal growth and long‐term cardiometabolic functions.^45^

As in humans, the adverse effects of maternal undernutrition upon offspring tend to develop with ageing. For example, rats exposed to low protein diets during foetal life can maintain normal renal function until they are 9 months old (approximately 40% of lifespan) but thereafter function declines more rapidly than in control rats and many males subject to maternal protein restriction die as a result of renal failure.^53^, ^54^ The same animals exhibit enhanced homeostatic responses to glucose loads as young adults but, at 18 months old, are insulin resistant, whereas control animals rarely show this impairment.^55^, ^56^. Consistent with the lifecourse view of health and disease, responses to nutritional challenges in adult life are modified by the foetal nutritional experience. Rats whose mothers were severely food restricted in pregnancy have an exaggerated response to a hypercaloric diet as adults, becoming more obese and metabolically impaired than offspring of mothers who were fed their normal diet.^57^ Genetically modified mice with a diet‐dependent predisposition to coronary heart disease had a greater atherogenic response to a high cholesterol diet if exposed to maternal protein restriction *in utero*.^58^

## MATERNAL OBESITY AND THE PROGRAMMING OF DISEASE

With respect to investigating the associations between maternal undernutrition and programming of disease, experimental animal studies proved to be a useful follow‐up to the epidemiological observations, critically demonstrating the biological plausibility of the programming hypothesis and generating data relating to possible programming mechanisms. When considering possible links between exposure to maternal obesity and disease in adult life, the animal studies came largely before any amassing of compelling epidemiological evidence.

Obesity can be difficult to induce in experimental animal species as rodents, in particular, are better able than humans to regulate their energy balance.^59^ Rats and mice are the species of choice for experimental models, and many studies rely on feeding diets high in fat and sugar to increase adiposity. Studies of this nature have shown that obesity in pregnancy can programme glucose intolerance, dyslipidaemia and elevated blood pressure in offspring.^60^ However, these experiments may be confounded by the fact that the offspring are exposed directly to these diets in addition to the maternal adiposity during foetal development and the suckling period. To modify the maternal diets to increase fat and sugar content inevitably reduces intake of protein and micronutrients and this is known to have a programming impact in itself.^47^ It is important to appreciate that these diets are provided to animals as a homogenous, pelleted foodstuff and ingesting high quantities of sucrose or specific fatty acids may be directly responsible for programming effects through their bioactivity, rather than the interpretation that the effects are a result of maternal obesity.^59^

An alternative approach has been to induce obesity using cafeteria feeding. This involves rats being offered a constantly changing array of highly palatable human foods in addition to their baseline (low energy) rodent feed.^59^, ^61^, ^62^, ^63^, ^64^ Once obesity is established, the rats can be transferred to their lower energy food, or maintained on the cafeteria diet for pregnancy, and offspring can be kept with their mothers or cross‐fostered to mothers with a different dietary or weight status. In this way, the effects of obesity during pregnancy and lactation can be studied independently of direct dietary effects. This approach has shown that maternal obesity can programme brain development and behaviour, adiposity and glucose homeostasis in offspring.^63^, ^64^, ^65^, ^66^ Rats exposed to cafeteria diet *in utero* have an enhanced preference for high sugar and high fat foods when they are adults. In non‐human primates, maternal obesity has similar effects to that observed in rodents. In young Japanese macaques, the offspring of obese mothers fed a Western style diet exhibited impaired insulin sensitivity in muscle even before they were weaned.^67^ Hypersecretion of insulin by pancreatic islets of young macaques exposed to this Western diet *in utero* suggests that there is a programmed dysfunction of glucose metabolism that will deteriorate with ageing.^68^

In humans, it is well recognised that there is a strong genetic component to obesity. Individuals with one or two obese parents are more likely to be obese themselves and the heritability of an obese phenotype has been estimated to be as high as 70%. Whitaker *et al*.^69^ reported that the risk of obesity with one obese parent was doubled, but interestingly the relationship was stronger where it was the mother who was obese. Such observations do not only reflect genetic influences because children will generally share their environment with their parents and hence experience the same dietary and behavioural drivers of excessive fat deposition. Exploration of a possible programming basis for this was initially stuck on the idea that low birthweight was a driver of later disease, as discussed earlier in this review. Studies showed that birth anthropometry was predictive of later obesity. A longitudinal follow‐up of the 1956 UK Birth Cohort found a J‐shaped relationship between birthweight and BMI in 33‐year‐old men and this appeared to be heavily driven by maternal but not paternal weight.^70^ A Finnish study found that the risk of abdominal obesity was greater in young adults who had been born small‐for‐gestational age.^71^ Generally, the evidence supported the view that obesity in adolescence and adulthood was predicted either by low birth weight or being a large baby at birth.^72^ These observations of indirect relationships between early life exposures, with birthweight as a proxy are, however, somewhat obsolete given there are more recent reports of direct associations between maternal BMI and adverse health indicators and outcomes in offspring.

The Growing Up Today study has followed up approximately 15,000 children of women who participated in the well‐characterised US Nurses Health Study.^73^ When followed up in adolescence, those who had been exposed to GDM during foetal development were more likely to be overweight. The analysis indicated an independent influence of maternal BMI in this relationship.^73^ If mothers maintained a healthy weight before pregnancy and engaged in a healthy dietary pattern, 150 min or more of moderate/vigorous exercise and avoided smoking, then their children were less likely to be obese between 9 and 14 years.^74^ Maternal BMI between 18.5 and 24.9 kg m^–2^ was associated with a lower risk of childhood obesity (odds ratio [OR] = 0.44, 95% confidence interval [CI] = 0.39–0.50 relative to higher BMI range) and maternal BMI was the strongest predictor of childhood weight outcomes. Other studies have similarly indicated that adiposity is greater in children whose mothers were living with obesity.^75^, ^76^, ^77^ A follow‐up of Thai 19–22 year olds found a 25% greater risk of obesity for every 1 kg m^–2^ increase in maternal BMI. The risk of offspring obesity among the children of mothers with BMI in the obese range was 17‐fold higher than in children of mothers of ideal weight.^78^

Of far greater significance are the observations that maternal obesity has a long‐term impact on metabolic function and disease outcomes. Some of these have been derived from record linkage studies of very large populations. Follow‐up of 2.23 million Swedish births between 1992 and 2016 found that diagnosis of cardiovascular disease between the ages of 1 and 25 years was more likely in those whose mothers had been obese in pregnancy than it was in those whose mothers had been of ideal weight.^79^ The risk was graded so that, although those whose mothers had BMI between 30 and 34.9 kg m^–2^ were 16% more likely to have cardiovascular disease, this increased to 2.51‐fold if maternal BMI was over 40 kg m^–2^. Tan *et al*.^77^ found that elevated cardiovascular disease risk factors (raised blood pressure, dyslipidaemia) were present in 13‐year‐old children of mothers who were overweight or obese relative to children of mothers of ideal weight. Follow‐up of Finnish men and women born between 1934 and 1944 found that those whose mothers had had a BMI > 28 kg m^–2^ in pregnancy were at greater risk of cardiovascular disease. Men were more prone to coronary heart disease and women to stroke.^80^ Reynolds *et al*.^81^ showed that, among 37,709 34–61‐year‐olds, all‐cause mortality was greater in those whose mothers had been obese than in those whose mothers had been of idea weight. Offspring of mothers with BMI > 30 kg m^–2^ were also more likely to have had a hospital admission with cardiovascular disease (OR = 1.29, 95% CI = 1.06–1.57).

Exposure to maternal obesity is associated with metabolic dysfunction. Boney *et al*.^82^ reported that 11‐year‐old children were at increased risk of developing the metabolic syndrome if born to mothers living with obesity. A similarly elevated risk of insulin resistance was observable in young men and, to a lesser extent, women if their mothers were obese,^76^ whereas Bucci *et al*.^83^ reported muscular insulin sensitivity was impaired in frail elderly men (average age 72 years) whose mothers had been obese. Follow‐up of men and women born in Helsinki in the 1930s and 40 s demonstrated that women whose mothers were of higher BMI were at greater risk of developing type 2 diabetes as adults.^80^ In the same way, record linkage of 118,201 Aberdeen births (1950–2011) to the Scottish diabetes register revealed that offspring of overweight (OR = 1.39, 95% CI = 1.06–1.83) and obese (OR = 3.8, 95% CI = 2.33–5.06) mothers were at markedly elevated risk of type 2 diabetes.^84^ There is also evidence that maternal obesity increases the risk of type 1 diabetes as among more than 1.26 million Swedish children born between 1992 and 2004, obesity in pregnancy predicted a type 1 diabetes diagnosis (OR = 1.33, 95% CI = 1.2–1.48).^85^ Similarly, analysis of data relating to the births of children who were subsequently hospitalised with type‐1 diabetes found that maternal BMI > 30 kg m^–2^ was a significant risk factor (OR = 1.29, 95% CI = 1.01–1.64).^86^ To some extent, this relationship could be explained by the association of maternal obesity with higher birthweight because there is an association between higher weight at birth and type 1 diabetes.^87^ Alternatively, it may be that maternal obesity is a driver of autoimmune damage to the infant pancreas. Analysis of blood markers of islet autoimmunity in neonates found that maternal obesity and gestational weight gain over 15 kg were associated with an autoimmune profile,^88^ although other studies have not confirmed this observation.^89^ Further studies suggest that maternal obesity may programme renal development and function^90^ and asthma.^91^

A number of studies are suggestive of programming effects of maternal obesity and/or obesogenic diets on appetite and food preferences in humans. A preference for a higher carbohydrate intake was observed in adult men, whose mothers were obese in pregnancy.^92^ Follow‐ups of the Avon Longitudinal Study of Parents and Children found that, at age 10 years, dietary choices were strongly related to those of mothers prepregnancy. There was no evidence of any paternal influence on children's food choice, and the relationship between childhood feeding and mother's postnatal behaviours was less marked. This supports the idea that appetite regulation is programmed *in utero*.^93^ In the same cohort, unhealthy maternal behaviours including consumption of ‘junk’ food in pregnancy was associated with fat mass in 15‐year‐old children, again with no paternal influence.^94^ Wardle *et al*.^95^ found that, among lean children with overweight or obese parents, there was a higher preference for fatty foods in taste tests and an ‘overeating’ eating style. Although the study did not split the cohort dependent on whether the mothers or fathers were obese, the average BMI of the mothers in the study was 36 kg m^–2^, whereas it was only 29 kg m^–2^ for the fathers. The data add to the view that maternal obesity determines offspring feeding behaviour in humans, as it does in experimental animals.^66^, ^96^, ^97^

## MECHANISTIC PERSPECTIVES ON PROGRAMMING BY OBESITY

Many putative mechanisms have been suggested to explain how maternal nutritional status during pregnancy can programme disease risk in the exposed offspring. For any programming to take place, there needs to be some signal, or signals, of the maternal environment to the foetus. This signal then has to be recognised and elicit a response. There is a lot of debate about the process of recognition to initiate the response, with many researchers suggesting that maternal nutritional status elicits changes to the foetal epigenome and thereby sets in train long‐term physiological adaptations, although the evidence for this is, as yet, not wholly convincing. The nature of the response to the maternal environment is somewhat easier to determine and one of the simplest mechanisms that can explain how variation in maternal nutritional status (including obesity) brings about changes in foetal anatomy and physiology involves the process of tissue remodelling. This rests on the idea that changes to the numbers of cells or the type of cells present within a tissue will reshape the morphology of that tissue and could have profound effects upon organ function.^6^

All organs and tissues are derived from small populations of embryonic progenitor cell lines, which go through waves of rapid cell proliferation and differentiation to achieve their development before parturition. An adverse maternal environment during these critical periods can effectively prevent formation of an optimal number of specialised structures (i.e. remodelling the genetically determined pattern) and limit the functional capacity of the mature organ. There is extensive evidence from animal studies of maternal undernutrition which demonstrates remodelling takes place in response to adverse conditions in a range of organs, including the kidneys, brain and pancreas^98^, ^99^, ^100^ This remodelling appears to underpin foetal programming of renal disease, appetite regulation and impaired metabolic regulation. Although harder to demonstrate in humans, there is evidence of associations between low birthweight and renal structure.^101^, ^102^, ^103^ The evidence base for tissue remodelling in response to maternal obesity is more limited but, in rodents, there is evidence that offspring of obese mothers fed a cafeteria diet prior to pregnancy also have altered renal structure (lower nephron number; A. Akyol and S. Langley‐Evans, unpublished data). Interestingly, ultrasound examination of the kidneys of infants who mothers were obese indigenous Australians indicated that they had lower kidney volume, consistent with having been remodelled.^90^

**Figure 5:**
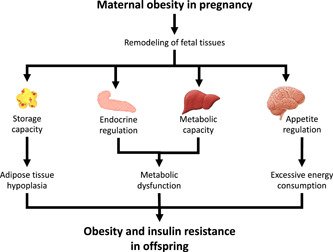
Remodelling of the structures of specific tissues in foetal life may explain how maternal obesity programmes offspring adiposity and metabolic function

Modifying the numbers and types of cells present within a tissue will have a range of consequences and the knock‐on effects on metabolic and physiological regulation will establish a predisposition for non‐communicable disease. This will not manifest as disease in childhood, instead being revealed when the individual undergoes metabolic or physiological challenge, or as tissue functions naturally deteriorate with age. Alterations to the profile of cell types present within a tissue may also modify the capacity of a tissue to produce or respond to hormones, alter gene expression or interfere with cell signaling pathways. Some of these changes may have very localised effects, simply impacting upon the function of a particular tissue, but others could disrupt regulation throughout the body. The epidemiological evidence that points to an association between maternal obesity and later disease in humans is well matched with the evidence from animal studies, and both point to disruption of metabolic regulation at the whole‐body level. As shown in Figure 5, this may result from remodelling of multiple tissues. Remodelling of adipose tissue so that there are fewer cells may underpin the observed propensity for offspring of women with high BMI to become obese because adipose tissue dysfunction impacts both the storage capacity of the tissue and regulation of metabolism by adipokines. Insulin resistance and the reported type 1 and type 2 diabetes in individuals exposed to obesity in foetal life could be explained by pancreatic remodelling and programming of liver structure could contribute to a number of metabolic anomalies including the dyslipidaemia reported by Tan *et al*.^77^ Remodelling of the hypothalamus has been reported as an outcome of maternal protein restriction in rats. If the tissue were also sensitive to maternal obesity, then the impact on whole‐body homeostasis could be profound. Evidence from rodent studies suggests maternal obesity during lactation does have an impact on hippocampal and hypothalamic neurotransmitter production, with consequent effects on behaviour and feeding.^65^, ^66^, ^104^ The observations that men who had obese mothers have a greater preference for carbohydrates^92^ and that children's food preferences follow their mother's prepregnancy behaviours but not their father's,^93^ may indicate that the same mechanisms could operate in humans.

**Figure 6:**
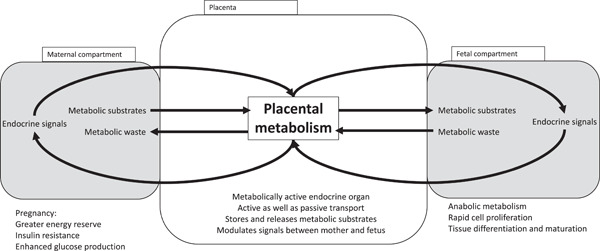
The placenta must mediate the signal of maternal nutritional status to the foetus

Tissue remodelling provides a route through which the adverse developmental environment of maternal obesity can programme offspring health, but does not explain how the foetal tissues receive signals of that environment. Whatever the programming stimulus or insult is, there is little doubt that it is mediated via the placenta. As shown in Figure 6, the placenta is not a passive facilitator of movement of oxygen, substrates and metabolic waste products between maternal and foetal compartments. It is a metabolically active tissue that generates substrates for the foetus and is a source of hormones and growth factors. All signals between mother and foetus are subject to modulation by placental activity.

The impact of maternal obesity on placentation is demonstrated by the greater risk of pre‐eclampsia^104^ in obese women. In pre‐eclampsia, inflammatory processes and oxidative injury leads to arterial dysfunction and breakdown of transport capacity.^105^ It is likely that the condition is the extreme endpoint of damaging impacts of maternal obesity on placental integrity and function. This probably has adverse programming effects on foetal development. Histopathological analyses of placentas from obese women show evidence of inflammatory processes and under‐perfusion, even in the absence of pre‐eclampsia.^106^ As early as the first trimester, obesity alters the expression of cell cycle regulatory genes in the placenta, which may impact on further placental growth and development and the capacity to maintain function at later stages of pregnancy.^107^ Among the hormones secreted by the placenta are leptin and adiponectin. These adipokines influence the development of adipose tissue in the foetus. Leptin also modulates the formation of the homeostatic endocrine axes in the foetal brain. Measurements of adipokine concentrations in cord blood at birth has shown elevated concentrations with maternal obesity.^108^

In addition to changes in the expression and release of endocrine signals, obesity impacts on fatty acid metabolism in the placenta. Altered expression of transcription factors and regulatory genes, including peroxisome proliferator activated receptor gamma coactivator 1 and carnitine palmitoyltransferase 1_alpha_ will impact on both lipid and carbohydrate metabolism and has been observed alongside elevated low‐density lipoprotein‐cholesterol and lower high‐density lipoprotein‐cholesterol concentrations in cord blood of foetuses exposed to maternal obesity.^109^ Similarly, the observation that expression of genes that regulate placental cholesterol transport is related to maternal BMI suggests that cholesterol handling is disrupted by maternal obesity.^110^ This may promote atherogenesis in the placental vessels (associated with pre‐eclampsia) and disrupt steroid hormone production. Obesity impacts upon fatty acid transport by the placenta and promotes an inflammatory response.^111^ The capacity of the placenta to store fatty acids is limited with obesity, resulting in greater mobilisation into the foetal compartment.^112^

Clues with respect to the mechanism of programming by maternal obesity may be gained from studies of GDM because the long‐term health of offspring exposed to GDM is largely the same as that observed with maternal obesity, although obesity can occur without GDM and vice versa. As early as 2 years of age, GDM offspring exhibit markedly greater risk of obesity^113^ and this persists into childhood.^114^, ^115^, ^116^ Dabelea *et al*.^117^ followed up sibling pairs where one of the pair had been exposed to GDM and the other had not. Among people in their early 20s, those who had experienced GDM in foetal life had a BMI on average 2.6 kg m^–2^ greater than unexposed siblings.^117^ Alongside greater risk of obesity, offspring of GDM‐affected pregnancies are at greater risk of metabolic disorders. Damm *et al*.^118^ reported a two‐fold greater risk of obesity in adults exposed to GDM *in utero*, accompanied by an eight‐fold greater risk of prediabetes and diabetes than in the background population. The adverse effects of exposure to GDM may be much broader, with, for example, reports of greater prevalence of psychiatric disorders in adults whose mothers had the condition in pregnancy.^119^

A simple explanation of how GDM and possibly maternal obesity provide the insult that programmes long‐term consequences for the exposed offspring is that an excess of energy substrates reaches the foetal compartment. The conventional wisdom is that this is the cause of macrosomia in GDM pregnancies because the foetus is hyperinsulinaemic and the insulin resistance of the mother drives glucose and lipids across the placenta.^8^ However, this is an over‐simplification because, similar to obesity, GDM has a broad impact on the placenta that will bring other factors into play. Widespread morphological changes including hypervascularisation and an increase in placental size and thickness are proposed to be a compensatory response to GDM which will preserve placental perfusion.^120^ There is also an increase in placental inflammation.^121^ Several defects of placental metabolism and function have been reported with GDM, including a reduction in iron transport^122^ and changes to lipid metabolism.^123^ With GDM, the placenta accumulates elevated concentrations of saturated fatty acids, with reduced transport of mono‐ and polyunsaturated fatty acids to the foetus.^123^

Although it is clear that the basic mechanisms driving the programming of health and disease by maternal obesity involve signalling across the placenta and a foetal tissue response at the level of gene and protein expression, the precise nature of the maternal signal and the foetal response in humans remain unknown. Identifying the mechanism is a high priority because, without this understanding, any intervention to prevent the long‐term consequences of maternal obesity will remain solely dependent upon health education and behaviour change strategies. Experience suggests that these have limited efficacy at the population level.

## IMPLICATIONS FOR THE FUTURE

**Figure 7:**
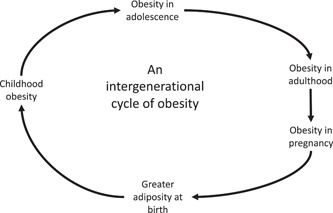
A transgenerational cycle of obesity and related disorders

The global obesity crisis will have profound consequences for the health of populations for decades to come. Obesity in adults is well recognised as a modifiable risk factor for type 2 diabetes, cardiovascular disease and many types of cancer. The evidence presented above would also suggest that the increasing numbers of individuals exposed to maternal overweight and obesity are themselves at greater risk of becoming obese and the associated cardiometabolic disorders. They will, in turn, be exposing their children to obesity *in utero*. There is a significant risk that a transgenerational cycle of obesity will be, or has already been, established (Figure 7). Such a cycle would have consequences for public health over a century or more unless effective means can be found to break it. Importantly, as obesity rates increase most rapidly in the populations of the global south, there is a risk of an explosion of metabolic disease on an unimaginable scale in nations ill‐equipped to deal with it.

Breaking such a cycle is a public health challenge of colossal complexity. The mode of intervention must be multifactorial, comprising locally tailored, culturally sensitive community education, widespread screening for predisease and investment in preventive health services. In short, a global shift in food cultures and living environments is necessary. Achieving this is unlikely, but, as the global focus moves towards sustainability, there may be opportunities to make inroads. The timing of interventions to break the transgenerational obesity cycle also needs to be considered in a more holistic manner. It is simple to think that the antenatal period is the key window for intervention. Limiting gestational weight gain and promoting a return to prepregnancy weight in the postpartum period will have many benefits. Pregnancy is perceived as a teachable moment when women are more open to public health messages and willing to make lifestyle changes,^8^, ^124^ although numerous large‐scale trials show limited efficacy of, and high resistance to pregnancy‐focused interventions.^125^, ^126^ The most effective approaches to managing weight gain in pregnancy appear to rely on more personalised interventions that are supported by eHealth packages and health professionals who have received appropriate training.^8^, ^127^, ^128^ Midwives, in particular, can find it difficult to engage with women about excess weight gain^129^, ^130^ but may find it useful to have an understanding of the transgenerational consequences of antenatal obesity as they frame their conversations with women.

Recommendations on antenatal weight management are heavily focused on women making changes to diet and lifestyle before they conceive.^8^ For women with extreme obesity, this might involve bariatric surgery and a number of studies demonstrate that women who achieve large weight loss through surgery have healthy pregnancies with reduced risk of complications and good outcomes.^131^, ^132^, ^133^ There is an emerging literature on the effects this weight loss may have on the long‐term health of babies born after weight loss. Smith *et al*.^134^ compared siblings whose mothers had undergone bariatric surgery, examining health indices in those born before and after the surgery. Individuals born after weight loss surgery were born with lower birthweight and were markedly less likely to be obese than their siblings born before the surgery. There was also evidence of better insulin sensitivity, lower concentrations of inflammatory markers and adipokine concentrations that were more consistent with metabolic health.^134^ However, the study only considered 49 sibling pairs and the ages of the subjects varied widely (2.6–26 years of age). The systematic review of Dunford and Sangster^135^ concluded that prepregnancy weight loss results in lower body fatness and improved insulin sensitivity in children born after weight loss compared to before, and suggested that changes to DNA methylation may play a role in this. A study of 31 sibling pairs noted differential DNA methylation of genes associated with insulin receptor signalling and type‐2 diabetes risk.^136^ However, because the study was small and the significance of methylation differences in whole blood samples is debatable, inferring a mechanism of programming from this is premature. It is hopeful, however, that action to address weight problems before pregnancy can prevent maternal programming of adverse health in the developing foetus. A number of trials are now underway aiming to address the impact of major weight loss on long‐term health and wellbeing.^137^, ^138^

Just as health at any stage of life is dependent upon the outcomes of gene–environment interactions at all preceding life stages, there are also opportunities to intervene and break the programmed trajectory during childhood. The literature that explores the tracking of obesity from childhood to adulthood indicates that the obese child is not predestined to become an obese adult, although obesity in adolescence does appear to track strongly to the adult years.^139^, ^140^ This highlights that the childhood years are a key time to address overweight and obesity that may have been programmed *in utero*. Importantly, the evidence shows that early intervention to reverse excessive weight in childhood removes any residual metabolic risk, such that the obese child who becomes a lean adult is at no cardiometabolic disadvantage.^139^, ^140^

Choices about infant feeding methods may represent the first point in the postnatal period when the impact of being an obese mother may be ameliorated. Systematic reviews and meta‐analyses indicate that breastfeeding reduces the risk of childhood and adult obesity, with exclusive breastfeeding and breastfeeding for a longer period (up to 12 months) having greater benefits.^141^ Horta *et al*.^142^ showed that breastfeeding was protective against overweight and obesity in both childhood (OR = 0.74, 95% CI = 0.68–0.79) and in adults (OR = 0.88, 95% CI = 0.82–0.94). The greater risk of overweight seen in formula fed infants could result from the higher protein content of formula milks,^143^ but it is also clear that breastfeeding brings advantages beyond just the milk composition. Demand‐led feeding, for example, will be associated with the normal development of satiety pathways and appetite regulation, and milk contains a range of non‐nutrient components. These include the appetite regulatory hormones leptin, adiponectin, resistin and ghrelin,^144^ which may play a key role in establishment of appetite control in the infant hypothalamus.^145^

Although breastfeeding may represent a means of compensating against exposure to obesity *in utero*, little is known about how obesity changes the composition (nutrient and hormone) of human milk and whether breastfeeding by an obese mother carries the same advantages as reported for the full breastfeeding population. Studies in rodents have identified that cafeteria feeding during lactation can programme offspring feeding and other behaviours, suggesting that milk may carry adverse programming signals.^65^, ^66^, ^146^ However, the immaturity of rat pups at birth makes them very different to human infants, and so the same milk‐related cues may not apply in the development of the human infant brain. Human milk is considered to be a highly dynamic food, with its composition changing according to stage of development, as well as in response to diet and time of day, and even varying between breasts in the same woman. However, much of the literature on milk composition is old and features poorly designed, small studies and little is known about how milk composition varies in response to acute changes in diet and what impact maternal adiposity may have. Leghi *et al*.^147^ reported that concentrations of macronutrients in milk showed little variation over a 3‐week‐period. Ward *et al*.^148^ found considerable diurnal variation in composition. Acutely increasing maternal fat consumption did not impact on macronutrients in milk over a 12‐h period, whereas, in contrast, an increase in sugar intake resulted in a rapid increase in milk triglycerides.^148^ A lot more research is required to understand what type of diet may be optimal for the production of an anti‐obesogenic milk profile by women and how this may vary between women of ideal weight and those who are overweight.

The introduction of complementary foods (weaning) is another point in time where decisions may have long‐term benefits for further health. The timing of weaning is considered to play an important role and, as described above, maintaining breastfeeding throughout the process prolongs exposure to human milk and the associated beneficial factors. There is evidence that very early introduction of solids (before 4 months) or delaying to beyond 6 months may increase the risk of childhood overweight.^149^ The inclusion of foods rich in protein appears advantageous in terms of infant growth and body composition but, if used in complementary feeding between 2 and 12 months, the risk of overweight in childhood is increased.^150^ There is a literature that considers feeding style during weaning, with some researchers advocating that a baby‐led weaning approach, rather than a parent‐led spoon‐feeding approach, reduces risk of later obesity by allowing the infant to self‐regulate intake and programme the development of satiety centres in the hypothalamus, which are not mature at birth. However, there is no significant evidence that there is a robust effect, and baby‐led weaned infants may in fact self‐select a diet that is high in sugars.^151^, ^152^, ^153^

To effectively meet the challenge of a transgenerational cycle of obesity and metabolic disorders, a multifaceted approach will be necessary. This needs to target: infants and children to promote healthy eating and lifestyles; adolescents to reinforce those messages before they become reproductively active; pregnant women to optimise nutrition, control weight gain and prevent GDM; and the postpartum period to promote a return to prepregnancy weight and facilitate long‐term breastfeeding.^154^ The emergence of evidence that paternal factors can also programme cardiometabolic health in offspring via semen‐related factors means that boys as well as girls need to be the focus of optimal health behaviours for parenting.^155^, ^156^ The global increase in obesity among children and adults is a public health concern with the potential to have consequences over many generations. The growing understanding that excessive adiposity in pregnancy can threaten both the immediate and long‐term health outcomes for the developing foetus should act as a stimulus for action across the world. Improving the nutrition and understanding of young people aiming to optimise their reproductive fitness will be a considerable challenge in the face of other societal and population health issues, but should be regarded as a high priority.

## AUTHOR CONTRIBUTIONS

Simon C. Langley‐Evans is responsible for the inception and writing of this review.

## ACKNOWLEDGEMENTS

No funding was received.

## CONFLICTS OF INTEREST

The author declares that there are no conflicts of interest.

## TRANSPARENCY DECLARATION

The lead author affirms that this manuscript is an honest, accurate and transparent account of the study being reported.

## References

[1] Doll R , Peto R . Cigarette smoking and bronchial carcinoma: dose and time relationships among regular smokers and lifelong non‐smokers. J Epidemiol Community Health. 1978;32:303–13. 744822 10.1136/jech.32.4.303 PMC1060963

[2] Keys A , Anderson JT , Grande F . Serum cholesterol response to changes in the diet: II. The effect of cholesterol in the diet. Metabolism. 1965;14:759–65. 25286460 10.1016/0026-0495(65)90002-8

[3] Goldstein JL , Brown MS . The low‐density lipoprotein pathway and its relation to atherosclerosis. Annu Rev Biochem. 1977;46:897–930. 197883 10.1146/annurev.bi.46.070177.004341

[4] Münzel T , Sørensen M , Lelieveld J , Hahad O , Al‐Kindi S , Nieuwenhuijsen M , et al. Heart healthy cities: genetics loads the gun but the environment pulls the trigger. Eur Heart J. 2021;42:2422–38. 34005032 10.1093/eurheartj/ehab235 PMC8248996

[5] Calder PC , Carding SR , Christopher G , Kuh D , Langley‐Evans SC , McNulty H . A holistic approach to healthy ageing: how can people live longer, healthier lives? J Hum Nutr Diet. 2018;31:439–50. 29862589 10.1111/jhn.12566

[6] Langley‐Evans SC . Nutrition in early life and the programming of adult disease: a review. J Hum Nutr Diet. 2015;28(Suppl 1):1–14. 10.1111/jhn.12212 24479490

[7] Ritchie H , Roser M ‘Obesity’. 2017 [cited 2022 February 14]. Available from: https://ourworldindata.org/obesity

[8] Langley‐Evans SC , Ellis S . Overweight, obesity and excessive weight gain in pregnancy as risk factors for adverse pregnancy outcomes. J Hum Nutr Diet. 2022;35:250–64. 35239212 10.1111/jhn.12999 PMC9311414

[9] Institute of Medicine . Weight Gain during pregnancy: reexamining the guidelines. Washington, DC: National Academies Press; 2009. 20669500

[10] Pan Y , Zhang S , Wang Q , Shen H , Zhang Y , Li Y , et al. Investigating the association between prepregnancy body mass index and adverse pregnancy outcomes: a large cohort study of 536 098 Chinese pregnant women in rural China. BMJ Open. 2016;6:e011227. 10.1136/bmjopen-2016-011227 PMC4964214 27439613

[11] Aune D , Saugstad OD , Henriksen T , Tonstad S . Maternal body mass index and the risk of fetal death, stillbirth, and infant death: a systematic review and meta‐analysis. JAMA. 2014;311:1536–46. 24737366 10.1001/jama.2014.2269

[12] Platner MH , Ackerman CM , Howland RE , Illuzzi J , Reddy UM , Bourjeily G , et al. Severe maternal morbidity and mortality during delivery hospitalization of class I, II, III, and super obese women. Am J Obstet Gynecol MFM. 2021;3:100420. 34157439 10.1016/j.ajogmf.2021.100420 PMC9667816

[13] Denison FC , Norrie G , Graham B , Lynch J , Harper N , Reynolds RM . Increased maternal BMI is associated with an increased risk of minor complications during pregnancy with consequent cost implications. BJOG. 2009;116:1467–72. 19496775 10.1111/j.1471-0528.2009.02222.x

[14] Relph S , Guo Y , Harvey ALJ , Vieira MC , Corsi DJ , Gaudet LM , et al. Characteristics associated with uncomplicated pregnancies in women with obesity: a population‐based cohort study. BMC Pregnancy Childbirth. 2021;21:182. 33673827 10.1186/s12884-021-03663-2 PMC7934497

[15] Alwash SM , McIntyre HD , Mamun A . The association of general obesity, central obesity and visceral body fat with the risk of gestational diabetes mellitus: evidence from a systematic review and meta‐analysis. Obes Res Clin Pract. 2021;15:425–30. 34391692 10.1016/j.orcp.2021.07.005

[16] He XJ , Dai RX , Hu CL . Maternal prepregnancy overweight and obesity and the risk of preeclampsia: a meta‐analysis of cohort studies. Obes Res Clin Pract. 2020;14:27–33. 32035840 10.1016/j.orcp.2020.01.004

[17] Hadden DR . Prediabetes and the big baby. Diabetic Med. 2008;25:1–10. 10.1111/j.1464-5491.2007.02331.x 18199127

[18] Williams AJ , Marryat L , Frank J . Cohort study of high maternal body mass index and the risk of adverse pregnancy and delivery outcomes in Scotland. BMJ Open. 2020;10:e026168. 10.1136/bmjopen-2018-026168 PMC7045241 32086347

[19] Marchi J , Berg M , Dencker A , Olander EK , Begley C . Risks associated with obesity in pregnancy, for the mother and baby: a systematic review of reviews. Obes Rev. 2015;16:621–38. 26016557 10.1111/obr.12288

[20] Dalbye R , Gunnes N , Blix E . Maternal body mass index and risk of obstetric, maternal and neonatal outcomes: a cohort study of nulliparous women with spontaneous onset of labor. Acta Obstet Gynecol Scand. 2021;100:521–30. 33031566 10.1111/aogs.14017

[21] Osmond C , Barker DJ , Slattery JM . Risk of death from cardiovascular disease and chronic bronchitis determined by place of birth in England and Wales. J Epidemiol Community Health. 1990;44:139–41. 2370502 10.1136/jech.44.2.139 PMC1060622

[22] Barker DJ , Osmond C . Infant mortality, childhood nutrition, and ischaemic heart disease in England and Wales. Lancet. 1986;1:1077–81. 2871345 10.1016/s0140-6736(86)91340-1

[23] Barker DJ , Winter PD , Osmond C , Margetts B , Simmonds SJ . Weight in infancy and death from ischaemic heart disease. Lancet. 1989;2:577–80. 2570282 10.1016/s0140-6736(89)90710-1

[24] Leon DA , Lithell HO , Vâgerö D , Koupilová I , Mohsen R , Berglund L , et al. Reduced fetal growth rate and increased risk of death from ischaemic heart disease: cohort study of 15 000 Swedish men and women born 1915–29. BMJ. 1998;317:241–5. 9677213 10.1136/bmj.317.7153.241 PMC28614

[25] Rich‐Edwards JW , Stampfer MJ , Manson JE , Rosner B , Hankinson SE , Colditz GA , et al. Birth weight and risk of cardiovascular disease in a cohort of women followed up since 1976. BMJ. 1997;315:396–400. 9277603 10.1136/bmj.315.7105.396 PMC2127275

[26] Yajnik CS , Fall CH , Vaidya U , Pandit AN , Bavdekar A , Bhat DS , et al. Fetal growth and glucose and insulin metabolism in four‐year‐old Indian children. Diabet Med. 1995;12:330–6. 7600749 10.1111/j.1464-5491.1995.tb00487.x

[27] Barker DJ , Hales CN , Fall CH , Osmond C , Phipps K , Clark PM . Type 2 (non‐insulin‐dependent) diabetes mellitus, hypertension and hyperlipidaemia (syndrome X): relation to reduced fetal growth. Diabetologia. 1993;36:62–7. 8436255 10.1007/BF00399095

[28] Hales CN , Barker DJ , Clark PM , Cox LJ , Fall C , Osmond C , et al. Fetal and infant growth and impaired glucose tolerance at age 64. BMJ. 1991;303:1019–22. 1954451 10.1136/bmj.303.6809.1019 PMC1671766

[29] Whincup PH , Kaye SJ , Owen CG , Huxley R , Cook DG , Anazawa S , et al. Birth weight and risk of type 2 diabetes: a systematic review. JAMA. 2008;300:2886–97. 19109117 10.1001/jama.2008.886

[30] Carrington LJ , Langley‐Evans SC . Wheezing and eczema in relation to infant anthropometry: evidence of developmental programming of disease in childhood. Matern Child Nutr. 2006;2:51–61. 16881914 10.1111/j.1740-8709.2006.00036.x PMC6860805

[31] Lithell HO , McKeigue PM , Berglund L , Mohsen R , Lithell UB , Leon DA . Relation of size at birth to non‐insulin dependent diabetes and insulin concentrations in men aged 50‐60 years.BMJ. 1996;312:406–10. 8601111 10.1136/bmj.312.7028.406 PMC2350082

[32] Barker DJ , Martyn CN , Osmond C , Wield GA . Abnormal liver growth in utero and death from coronary heart disease. BMJ. 1995;310:703–4. 7711538 10.1136/bmj.310.6981.703 PMC2549097

[33] Phillips DI , Barker DJ , Hales CN , Hirst S , Osmond C . Thinness at birth and insulin resistance in adult life. Diabetologia. 1994;37:150–4. 8163048 10.1007/s001250050086

[34] Forsén T , Eriksson JG , Tuomilehto J , Osmond C , Barker DJ . Growth in utero and during childhood among women who develop coronary heart disease: longitudinal study. BMJ. 1999;319:1403–7. 10574856 10.1136/bmj.319.7222.1403 PMC28284

[35] Eriksson JG , Lindi V , Uusitupa M , Forsén TJ , Laakso M , Osmond C , et al. The effects of the Pro12Ala polymorphism of the peroxisome proliferator‐activated receptor‐gamma2 gene on insulin sensitivity and insulin metabolism interact with size at birth. Diabetes. 2002;51:2321–4. 12086968 10.2337/diabetes.51.7.2321

[36] Eriksson J , Lindi V , Uusitupa M , Forsén T , Laakso M , Osmond C , et al. The effects of the Pro12Ala polymorphism of the PPARgamma‐2 gene on lipid metabolism interact with body size at birth. Clin Genet. 2003;64:366–70. 12974743 10.1034/j.1399-0004.2003.00150.x

[37] Roseboom TJ , Painter RC , van Abeelen AF , Veenendaal MV , de Rooij SR . Hungry in the womb: what are the consequences? Lessons from the Dutch famine. Maturitas. 2011;70:141–5. 21802226 10.1016/j.maturitas.2011.06.017

[38] de Rooij SR , Roseboom TJ , Painter RC . Famines in the last 100 years: implications for diabetes. Curr Diab Rep. 2014;14:536. 25173690 10.1007/s11892-014-0536-7

[39] Meng R , Yu C , Guo Y , Bian Z , Si J , Nie J , et al. Early famine exposure and adult disease risk based on a 10‐year prospective study of Chinese adults. Heart. 2020;106:213–20. 31704783 10.1136/heartjnl-2019-315750 PMC6968949

[40] Liu J , Wang G , Wu Y , Guan Y , Luo Z , Zhao G , et al. Early‐life exposure to famine and risk of metabolic associated fatty liver disease in Chinese adults. Nutrients. 2021;13:4063. 34836318 10.3390/nu13114063 PMC8622729

[41] Wang B , Cheng J , Wan H , Wang Y , Zhang W , Chen Y , et al. Early‐life exposure to the Chinese famine, genetic susceptibility and the risk of type 2 diabetes in adulthood. Diabetologia. 2021;64:1766–74. 33885932 10.1007/s00125-021-05455-x

[42] Huang YQ , Liu L , Yu YL , Lo K , Chen CL , Huang JY , et al. The relationship between famine exposure in early life and left atrial enlargement in adulthood. J Hum Nutr Diet. 2021;34:356–64. 32830406 10.1111/jhn.12802

[43] Hales CN , Barker DJ . The thrifty phenotype hypothesis. Br Med Bull. 2001;60:5–20. 11809615 10.1093/bmb/60.1.5

[44] Bateson P , Gluckman P , Hanson M . The biology of developmental plasticity and the predictive adaptive response hypothesis. J Physiol. 2014;592:2357–68. 24882817 10.1113/jphysiol.2014.271460 PMC4048093

[45] Hattersley AT , Tooke JE . The fetal insulin hypothesis: an alternative explanation of the association of low birthweight with diabetes and vascular disease. Lancet. 1999;353:1789–92. 10348008 10.1016/S0140-6736(98)07546-1

[46] Warrington NM , Beaumont RN , Horikoshi M , Day FR , Helgeland Ø , Laurin C , et al. Maternal and fetal genetic effects on birth weight and their relevance to cardio‐metabolic risk factors. Nat Genet. 2019;51:804–14. 31043758 10.1038/s41588-019-0403-1 PMC6522365

[47] Langley‐Evans SC . Fetal programming of CVD and renal disease: animal models and mechanistic considerations. Proc Nutr Soc. 2013;72:317–25. 23312451 10.1017/S0029665112003035

[48] Inskip HM , Godfrey KM , Robinson SM , Law CM , Barker DJ , Cooper C , et al. Cohort profile: the Southampton Women's Survey. Int J Epidemiol. 2006;35:42–8. 16195252 10.1093/ije/dyi202 PMC4579566

[49] Soh SE , Tint MT , Gluckman PD , Godfrey KM , Rifkin‐Graboi A , Chan YH , et al. Cohort profile: Growing Up in Singapore Towards healthy Outcomes (GUSTO) birth cohort study. Int J Epidemiol. 2014;43:1401–9. 23912809 10.1093/ije/dyt125

[50] Oken E , Baccarelli AA , Gold DR , Kleinman KP , Litonjua AA , De Meo D , et al. Cohort profile: project viva. Int J Epidemiol. 2015;44:37–48. 24639442 10.1093/ije/dyu008 PMC4339753

[51] McMullen S , Mostyn A . Animal models for the study of the developmental origins of health and disease. Proc Nutr Soc. 2009;68:306–20. 19490740 10.1017/S0029665109001396

[52] Kuo AH , Li C , Li J , Huber HF , Nathanielsz PW , Clarke GD . Cardiac remodelling in a baboon model of intrauterine growth restriction mimics accelerated ageing. J Physiol. 2017;595:1093–110. 27988927 10.1113/JP272908 PMC5309359

[53] Joles JA , Sculley DV , Langley‐Evans SC . Proteinuria in aging rats due to low‐protein diet during mid‐gestation. J Dev Orig Health Dis. 2010;1(1):75–83. 25142934 10.1017/S2040174409990183

[54] Langley‐Evans SC , Sculley DV . The association between birthweight and longevity in the rat is complex and modulated by maternal protein intake during fetal life. FEBS Lett. 2006;580:4150–3. 16828754 10.1016/j.febslet.2006.06.062

[55] Bellinger L , Sculley DV , Langley‐Evans SC . Exposure to undernutrition in fetal life determines fat distribution, locomotor activity and food intake in ageing rats. Int J Obes (Lond). 2006;30:729–38. 16404403 10.1038/sj.ijo.0803205 PMC1865484

[56] Erhuma A , Salter AM , Sculley DV , Langley‐Evans SC , Bennett AJ . Prenatal exposure to a low‐protein diet programs disordered regulation of lipid metabolism in the aging rat. Am J Physiol Endocrinol Metab. 2007;292:E1702–14. 17299084 10.1152/ajpendo.00605.2006 PMC1890310

[57] Vickers MH , Breier BH , Cutfield WS , Hofman PL , Gluckman PD . Fetal origins of hyperphagia, obesity, and hypertension and postnatal amplification by hypercaloric nutrition. Am J Physiol Endocrinol Metab. 2000;279:E83–7. 10893326 10.1152/ajpendo.2000.279.1.E83

[58] Yates Z , Tarling EJ , Langley‐Evans SC , Salter AM . Maternal undernutrition programmes atherosclerosis in the ApoE*3‐Leiden mouse. Br J Nutr. 2009;101:1185–94. 18782462 10.1017/S0007114508066786 PMC2670275

[59] Sampey BP , Vanhoose AM , Winfield HM , Freemerman AJ , Muehlbauer MJ , Fueger PT , et al. Cafeteria diet is a robust model of human metabolic syndrome with liver and adipose inflammation: comparison to high‐fat diet. Obesity (Silver Spring). 2011;19(6):1109–17. 21331068 10.1038/oby.2011.18 PMC3130193

[60] Samuelsson AM , Matthews PA , Argenton M , Christie MR , McConnell JM , Jansen EH , et al. Diet‐induced obesity in female mice leads to offspring hyperphagia, adiposity, hypertension, and insulin resistance: a novel murine model of developmental programming. Hypertension. 2008;51:383–92. 18086952 10.1161/HYPERTENSIONAHA.107.101477

[61] Akyol A , McMullen S , Langley‐Evans SC . Glucose intolerance associated with early‐life exposure to maternal cafeteria feeding is dependent upon post‐weaning diet. Br J Nutr. 2012;107:964–78. 21861941 10.1017/S0007114511003916

[62] Vithayathil MA , Gugusheff JR , Ong ZY , Langley‐Evans SC , Gibson RA , Muhlhausler BS . Exposure to maternal cafeteria diets during the suckling period has greater effects on fat deposition and Sterol Regulatory Element Binding Protein‐1c (SREBP‐1c) gene expression in rodent offspring compared to exposure before birth. Nutr Metab (Lond). 2018;15:17. 29467799 10.1186/s12986-018-0253-3 PMC5815184

[63] George G , Draycott SAV , Muir R , Clifford B , Elmes MJ , Langley‐Evans SC . The impact of exposure to cafeteria diet during pregnancy or lactation on offspring growth and adiposity before weaning. Sci Rep. 2019;9:14173. 31578441 10.1038/s41598-019-50448-x PMC6775089

[64] George G , Draycott SAV , Muir R , Clifford B , Elmes MJ , Langley‐Evans SC . Exposure to maternal obesity during suckling outweighs in utero exposure in programming for post‐weaning adiposity and insulin resistance in rats. Sci Rep. 2019;9:10134. 31300679 10.1038/s41598-019-46518-9 PMC6626015

[65] Wright T , Langley‐Evans SC , Voigt JP . The impact of maternal cafeteria diet on anxiety‐related behaviour and exploration in the offspring. Physiol Behav. 2011;103:164–72. 21241725 10.1016/j.physbeh.2011.01.008

[66] Wright TM , Fone KC , Langley‐Evans SC , Voigt JP . Exposure to maternal consumption of cafeteria diet during the lactation period programmes feeding behaviour in the rat. Int J Dev Neurosci. 2011;29:785–93. 22004940 10.1016/j.ijdevneu.2011.09.007

[67] Campodonico‐Burnett W , Hetrick B , Wesolowski SR , Schenk S , Takahashi DL , Dean TA , et al. Maternal obesity and western‐style diet impair fetal and juvenile offspring skeletal muscle insulin‐stimulated glucose transport in nonhuman primates. Diabetes. 2020;69:1389–400. 32354857 10.2337/db19-1218 PMC7306120

[68] Elsakr JM , Dunn JC , Tennant K , Zhao SK , Kroeten K , Pasek RC , et al. Maternal Western‐style diet affects offspring islet composition and function in a non‐human primate model of maternal over‐nutrition. Mol Metab. 2019;25:73–82. 31036449 10.1016/j.molmet.2019.03.010 PMC6599455

[69] Whitaker RC , Wright JA , Pepe MS , Seidel KD , Dietz WH . Predicting obesity in young adulthood from childhood and parental obesity. N Engl J Med. 1997;337:869–73. 9302300 10.1056/NEJM199709253371301

[70] Parsons TJ , Power C , Manor O . Fetal and early life growth and body mass index from birth to early adulthood in 1958 British cohort: longitudinal study. BMJ. 2001;323:1331–5. 11739217 10.1136/bmj.323.7325.1331 PMC60670

[71] Laitinen J , Pietiläinen K , Wadsworth M , Sovio U , Järvelin MR . Predictors of abdominal obesity among 31‐y‐old men and women born in Northern Finland in 1966. Eur J Clin Nutr. 2004;58:180–90. 14679384 10.1038/sj.ejcn.1601765

[72] Catalano PM , Ehrenberg HM . The short‐ and long‐term implications of maternal obesity on the mother and her offspring. BJOG. 2006;113:1126–33. 16827826 10.1111/j.1471-0528.2006.00989.x

[73] Gillman MW , Rifas‐Shiman S , Berkey CS , Field AE , Colditz GA . Maternal gestational diabetes, birth weight, and adolescent obesity. Pediatrics. 2003;111:e221–6. 12612275 10.1542/peds.111.3.e221

[74] Dhana K , Haines J , Liu G , Zhang C , Wang X , Field AE , et al. Association between maternal adherence to healthy lifestyle practices and risk of obesity in offspring: results from two prospective cohort studies of mother‐child pairs in the United States. BMJ. 2018;362:k2486. 29973352 10.1136/bmj.k2486 PMC6031199

[75] Catalano PM , Presley L , Minium J , Hauguel‐de Mouzon S . Fetuses of obese mothers develop insulin resistance in utero. Diabetes Care. 2009;32:1076–80. 19460915 10.2337/dc08-2077 PMC2681036

[76] Mingrone G , Manco M , Mora ME , Guidone C , Iaconelli A , Gniuli D , et al. Influence of maternal obesity on insulin sensitivity and secretion in offspring. Diabetes Care. 2008;31:1872187–6. 10.2337/dc08-0432 PMC2518362 18535193

[77] Tan HC , Roberts J , Catov J , Krishnamurthy R , Shypailo R , Bacha F . Mother's pre‐pregnancy BMI is an important determinant of adverse cardiometabolic risk in childhood. Pediatr Diabetes. 2015;16:419–26. 25800542 10.1111/pedi.12273 PMC4534350

[78] Ounjaijean S , Wongthanee A , Kulprachakarn K , Rerkasem A , Pruenglampoo S , Mangklabruks A , et al. Higher maternal BMI early in pregnancy is associated with overweight and obesity in young adult offspring in Thailand. BMC Public Health. 2021;21:724. 33853557 10.1186/s12889-021-10678-z PMC8048216

[79] Razaz N , Villamor E , Muraca GM , Bonamy AE , Cnattingius S . Maternal obesity and risk of cardiovascular diseases in offspring: a population‐based cohort and sibling‐controlled study. Lancet Diabetes Endocrinol. 2020;8:572–81. 32559473 10.1016/S2213-8587(20)30151-0

[80] Eriksson JG , Sandboge S , Salonen MK , Kajantie E , Osmond C . Long‐term consequences of maternal overweight in pregnancy on offspring later health: findings from the Helsinki Birth Cohort Study. Ann Med. 2014;46:434–8. 24910160 10.3109/07853890.2014.919728

[81] Reynolds RM , Allan KM , Raja EA , Bhattacharya S , McNeill G , Hannaford PC , et al. Maternal obesity during pregnancy and premature mortality from cardiovascular event in adult offspring: follow‐up of 1 323 275 person years. BMJ. 2013;347:f4539. 23943697 10.1136/bmj.f4539 PMC3805484

[82] Boney CM , Verma A , Tucker R , Vohr BR . Metabolic syndrome in childhood: association with birth weight, maternal obesity, and gestational diabetes mellitus. Pediatrics. 2005;115:e290–6. 15741354 10.1542/peds.2004-1808

[83] Bucci M , Huovinen V , Guzzardi MA , Koskinen S , Raiko JR , Lipponen H , et al. Resistance training improves skeletal muscle insulin sensitivity in elderly offspring of overweight and obese mothers. Diabetologia. 2016;59:77–86. 26486356 10.1007/s00125-015-3780-8

[84] Lahti‐Pulkkinen M , Räikkönen K , Bhattacharya S , Reynolds RM . Maternal body mass index in pregnancy and mental disorders in adult offspring: a record linkage study in Aberdeen, Scotland. Sci Rep. 2021;11:15132. 34302021 10.1038/s41598-021-94511-y PMC8302653

[85] Hussen HI , Persson M , Moradi T . Maternal overweight and obesity are associated with increased risk of type 1 diabetes in offspring of parents without diabetes regardless of ethnicity. Diabetologia. 2015;58:1464–73. 25940642 10.1007/s00125-015-3580-1

[86] Arkkola T , Kautiainen S , Takkinen , Kenward MG , Nevalainen J , Uusitalo U , et al. Relationship of maternal weight status and weight gain rate during pregnancy to the development of advanced beta cell autoimmunity in the offspring: a prospective birth cohort study. Pediatr Diabetes. 2011;12:478–84. 21129139 10.1111/j.1399-5448.2010.00703.x

[87] Stene LC , Magnus P , Lie RT , Sovik O , Joner G . Birth weight and childhood onset type 1 diabetes: population based cohort study. BMJ. 2001;322:889–92. 11302899 10.1136/bmj.322.7291.889 PMC30582

[88] Rasmussen T , Stene LC , Samuelsen , Cinek O , Wetlesen T , Torjesen PA , et al. Maternal BMI before pregnancy, maternal weight gain during pregnancy, and risk of persistent positivity for multiple diabetes‐associated autoantibodies in children with the high‐risk HLA genotype: the MIDIA study. Diabetes Care. 2009;32:1904–06. 19592628 10.2337/dc09-0663 PMC2752934

[89] Arkkola T , Kautiainen S , Takkinen , Kenward MG , Nevalainen J , Uusitalo U , et al. Relationship of maternal weight status and weight gain rate during pregnancy to the development of advanced beta cell autoimmunity in the offspring: a prospective birth cohort study. Pediatr Diabetes. 2011;12:478–84. 21129139 10.1111/j.1399-5448.2010.00703.x

[90] Lee YQ , Lumbers ER , Oldmeadow C , Collins CE , Johnson V , Keogh L , et al. The relationship between maternal adiposity during pregnancy and fetal kidney development and kidney function in infants: the Gomeroi gaaynggal study. Physiol Rep. 2019;7:e14227. 31515958 10.14814/phy2.14227 PMC6742895

[91] Patel SP , Rodriguez A , Little MP , Elliott P , Pekkanen J , Hartikainen AL , et al. Associations between pre‐pregnancy obesity and asthma symptoms in adolescents. J Epidemiol Community Health. 2012;66:809–14. 21844604 10.1136/jech.2011.133777 PMC3412048

[92] Kaseva N , Vääräsmäki M , Matinolli HM , Sipola M , Tikanmäki M , Kanerva N , et al. Maternal pre‐pregnancy overweight and gestational diabetes and dietary intakes among young adult offspring. Nutr Diabetes. 2020;10:26. 32703940 10.1038/s41387-020-00129-w PMC7378069

[93] Brion MJ , Ness AR , Rogers I , Emmett P , Cribb V , Davey Smith G , et al. Maternal macronutrient and energy intakes in pregnancy and offspring intake at 10 y: exploring parental comparisons and prenatal effects. Am J Clin Nutr. 2010;91:748–56. 20053880 10.3945/ajcn.2009.28623 PMC2822901

[94] Leary SD , Lawlor DA , Davey Smith G , Brion MJ , Ness AR . Behavioural early‐life exposures and body composition at age 15 years. Nutr Diabetes. 2015;5:e150. 25664839 10.1038/nutd.2014.47 PMC4338416

[95] Wardle J , Guthrie C , Sanderson S , Birch L , Plomin R . Food and activity preferences in children of lean and obese parents. Int J Obes Relat Metab Disord. 2001;25:971–7. 11443494 10.1038/sj.ijo.0801661

[96] Bayol SA , Simbi BH , Stickland NC . A maternal cafeteria diet during gestation and lactation promotes adiposity and impairs skeletal muscle development and metabolism in rat offspring at weaning. J Physiol. 2005;567:951–61. 16020464 10.1113/jphysiol.2005.088989 PMC1474235

[97] Bayol SA , Farrington SJ , Stickland NC . A maternal ‘junk food’ diet in pregnancy and lactation promotes an exacerbated taste for ‘junk food’ and a greater propensity for obesity in rat offspring. Br J Nutr. 2007;98:843–51. 17697422 10.1017/S0007114507812037

[98] Langley‐Evans SC , Welham SJ , Jackson AA . Fetal exposure to a maternal low protein diet impairs nephrogenesis and promotes hypertension in the rat. Life Sci. 1999;64:965–74. 10201645 10.1016/s0024-3205(99)00022-3

[99] Plagemann A , Harder T , Rake A , Melchior K , Rohde W , Dörner G . Hypothalamic nuclei are malformed in weanling offspring of low protein malnourished rat dams. J Nutr. 2000;130:2582–9. 11015493 10.1093/jn/130.10.2582

[100] Snoeck A , Remacle C , Reusens B , Hoet JJ . Effect of a low protein diet during pregnancy on the fetal rat endocrine pancreas. Biol Neonate. 1990;57:107–18. 2178691 10.1159/000243170

[101] Lumbers ER , Kandasamy Y , Delforce SJ , Boyce AC , Gibson KJ , Pringle KG . Programming of renal development and chronic disease in adult life. Front Physiol. 2020;11:757. 32765290 10.3389/fphys.2020.00757 PMC7378775

[102] Hughson M , Farris AB , Douglas‐Denton R , Hoy WE , Bertram JF . Glomerular number and size in autopsy kidneys: the relationship to birth weight. Kidney Int. 2003;63:2113–22. 12753298 10.1046/j.1523-1755.2003.00018.x

[103] Luyckx VA , Perico N , Somaschini M , Manfellotto D , Valensise H , Cetin I , et al. A developmental approach to the prevention of hypertension and kidney disease: a report from the Low Birth Weight and Nephron Number Working Group. Lancet. 2017;390:424–8. 28284520 10.1016/S0140-6736(17)30576-7 PMC5884413

[104] Lamminpää R , Vehviläinen‐Julkunen K , Gissler M , Selander T , Heinonen S . Pregnancy outcomes of overweight and obese women aged 35 years or older ‐ a registry‐based study in Finland. Obes Res Clin Pract. 2016;10:133–42. 26054598 10.1016/j.orcp.2015.05.008

[105] Poston L . Endothelial dysfunction in pre‐eclampsia. Pharmacol Rep. 2006;58(Suppl):69–74. 17332674

[106] Brouwers L , Franx A , Vogelvang TE , Houben ML , van Rijn BB , Nikkels PG . Association of maternal prepregnancy body mass index with placental histopathological characteristics in uncomplicated term pregnancies. Pediatr Dev Pathol. 2019;22:45–52. 29969058 10.1177/1093526618785838 PMC6604681

[107] Hoch D , Bachbauer M , Pöchlauer C , Algaba‐Chueca F , Tandl V , Novakovic B , et al. Maternal obesity alters placental cell cycle regulators in the first trimester of human pregnancy: new insights for BRCA1. Int J Mol Sci. 2020;21:468. 10.3390/ijms21020468 PMC7014057 31940810

[108] Jaramillo‐Ospina Á , Castaño‐Moreno E , Muñoz‐Muñoz E , Krause BJ , Uauy R , Casanello P , et al. Maternal obesity is associated with higher cord blood adipokines in offspring most notably in females. J Pediatr Gastroenterol Nutr. 2021;73:264–70. 34016877 10.1097/MPG.0000000000003172

[109] Bucher M , Montaniel KRC , Myatt L , Weintraub S , Tavori H , Maloyan A . Dyslipidemia, insulin resistance, and impairment of placental metabolism in the offspring of obese mothers. J Dev Orig Health Dis. 2021;12:738–47. 33185172 10.1017/S2040174420001026 PMC8606174

[110] Draycott SAV , Daniel Z , Khan R , Muhlhausler BS , Elmes MJ , Langley‐Evans SC . Expression of cholesterol packaging and transport genes in human and rat placenta: impact of obesity and a high‐fat diet. J Dev Orig Health Dis. 2020;11:222–7. 31601282 10.1017/S2040174419000606

[111] Belcastro L , Ferreira CS , Saraiva MA , Mucci DB , Murgia A , Lai C , et al. Nutrients. 2021;13:2768. 34444927 10.3390/nu13082768 PMC8398812

[112] Hirschmugl B , Perazzolo S , Sengers BG , Lewis RM , Gruber M , Desoye G , et al. Placental mobilization of free fatty acids contributes to altered materno‐fetal transfer in obesity. Int J Obes (Lond). 2021;45:1114–23. 33637949 10.1038/s41366-021-00781-x PMC8081658

[113] Zou J , Yang Y , Wei Q , Zhang Y , Shi H . Longitudinal association of maternal pre‐pregnancy BMI and third‐trimester glycemia with early life growth of offspring: a prospective study among GDM‐negative pregnant women. Nutrients. 2021;13:3971. 34836226 10.3390/nu13113971 PMC8619788

[114] Norris T , Mansukoski L , Gilthorpe MS , Hamer M , Hardy R , Howe LD , et al. Early childhood weight gain: latent patterns and body composition outcomes. Paediatr Perinat Epidemiol. 2021;35:557–68. 33960515 10.1111/ppe.12754

[115] Loo EXL , Zhang Y , Yap QV , Yu G , Soh SE , Loy SL , et al. Comparative epidemiology of gestational diabetes in ethnic Chinese from Shanghai birth cohort and growing up in Singapore towards healthy outcomes cohort. BMC Pregnancy Childbirth. 2021;21:566. 34407778 10.1186/s12884-021-04036-5 PMC8375167

[116] Chivese T , Haynes MC , van Zyl H , Kyriacos U , Levitt NS , Norris SA . The influence of maternal blood glucose during pregnancy on weight outcomes at birth and preschool age in offspring exposed to hyperglycemia first detected during pregnancy, in a South African cohort. PLoS One. 2021;16:e0258894. 34673829 10.1371/journal.pone.0258894 PMC8530360

[117] Dabelea D , Hanson RL , Lindsay RS , Pettitt DJ , Imperatore G , Gabir MM , et al. Intrauterine exposure to diabetes conveys risks for type 2 diabetes and obesity: a study of discordant sibships. Diabetes. 2000;49:2208–11. 11118027 10.2337/diabetes.49.12.2208

[118] Damm P , Houshmand‐Oeregaard A , Kelstrup L , Lauenborg J , Mathiesen ER , Clausen TD . Gestational diabetes mellitus and long‐term consequences for mother and offspring: a view from Denmark. Diabetologia. 2016;59:1396–9. 27174368 10.1007/s00125-016-3985-5

[119] Nogueira Avelar E , Silva R , Yu Y , Liew Z , Vested A , Sørensen HT , et al. Associations of maternal diabetes during pregnancy with psychiatric disorders in offspring during the first 4 decades of life in a population‐based Danish birth cohort. JAMA Netw Open. 2021;4:e2128005. 34648013 10.1001/jamanetworkopen.2021.28005 PMC8517748

[120] Carrasco‐Wong I , Moller A , Giachini FR , Lima VV , Toledo F , Stojanova J , et al. Placental structure in gestational diabetes mellitus. Biochim Biophys Acta, Mol Basis Dis. 2020;1866:165535. 31442531 10.1016/j.bbadis.2019.165535

[121] Li YX , Long DL , Liu J , Qiu D , Wang J , Cheng X , et al. Gestational diabetes mellitus in women increased the risk of neonatal infection via inflammation and autophagy in the placenta. Medicine (Baltimore). 2020;99:e22152. 33019392 10.1097/MD.0000000000022152 PMC7535644

[122] Zaugg J , Melhem H , Huang X , Wegner M , Baumann M , Surbek D , et al. Gestational diabetes mellitus affects placental iron homeostasis: mechanism and clinical implications. FASEB J. 2020;34:7311–29. 32285992 10.1096/fj.201903054R

[123] Stirm L , Kovárová M , Perschbacher S , Michlmaier R , Fritsche L , Siegel‐Axel D , et al. BMI‐independent effects of gestational diabetes on human placenta. J Clin Endocrinol Metab. 2018;103:3299–309. 29931171 10.1210/jc.2018-00397

[124] Phelan S . Pregnancy: a ‘teachable moment’ for weight control and obesity prevention. Am J Obstet Gynecol. 2010;202(135):e1–8. 10.1016/j.ajog.2009.06.008 PMC2815033 19683692

[125] Dodd JM , McPhee AJ , Turnbull D , Yelland LN , Deussen AR , Grivell RM , et al. The effects of antenatal dietary and lifestyle advice for women who are overweight or obese on neonatal health outcomes: the LIMIT randomised trial. BMC Med. 2014;12:163. 25315325 10.1186/s12916-014-0163-9 PMC4194368

[126] Poston L , Bell R , Croker H , Flynn AC , Godfrey KM , Goff L , et al. Effect of a behavioural intervention in obese pregnant women (the UPBEAT study): a multicentre, randomised controlled trial. Lancet Diabetes Endocrinol. 2015;3:767–77. 26165396 10.1016/S2213-8587(15)00227-2

[127] Lawrence W , Vogel C , Strömmer S , Morris T , Treadgold B , Watson D , et al. How can we best use opportunities provided by routine maternity care to engage women in improving their diets and health? Matern Child Nutr. 2020;16:e12900. 31736283 10.1111/mcn.12900 PMC7038869

[128] Downs DS , Savage JS , Rivera DE , Pauley AM , Leonard KS , Hohman EE , et al. Adaptive, behavioral intervention impact on weight gain, physical activity, energy intake, and motivational determinants: results of a feasibility trial in pregnant women with overweight/obesity. J Behav Med. 2021;44:605–21. 33954853 10.1007/s10865-021-00227-9 PMC9764232

[129] Soltani H , Duxbury A , Rundle R , Marvin‐Dowle K . Dietary habits and supplementation practices of young women during pregnancy: an online cross‐sectional survey of young mothers and health care professionals. BMC Nutr. 2017;3:19. 32153801 10.1186/s40795-017-0137-3 PMC7050751

[130] Heslehurst N , Lang R , Rankin J , Wilkinson HR , Summerbell CD . Obesity in pregnancy: a study of the impact of maternal obesity on NHS maternity services. BJOG. 2007;114:334–42. 17261124 10.1111/j.1471-0528.2006.01230.x

[131] Maslin K , Douek I , Greenslade B , Shawe J . Nutritional and perinatal outcomes of pregnant women with a history of bariatric surgery: a case series from a UK centre. J Hum Nutr Diet. 2020;33:386–95. 31765078 10.1111/jhn.12718

[132] Johansson K , Cnattingius S , Näslund I , Roos N , Trolle Lagerros Y , Granath F , et al. Outcomes of pregnancy after bariatric surgery. N Engl J Med. 2015;372(9):814–24. 25714159 10.1056/NEJMoa1405789

[133] Galazis N , Docheva N , Simillis C , Nicolaides KH . Maternal and neonatal outcomes in women undergoing bariatric surgery: a systematic review and meta‐analysis. Eur J Obstet Gynecol Reprod Biol. 2014;181:45–53. 25126981 10.1016/j.ejogrb.2014.07.015

[134] Smith J , Cianflone K , Biron S , Hould FS , Lebel S , Marceau S , et al. Effects of maternal surgical weight loss in mothers on intergenerational transmission of obesity. J Clin Endocrinol Metab. 2009;94:4275–83. 19820018 10.1210/jc.2009-0709

[135] Dunford AR , Sangster JM . Maternal and paternal periconceptional nutrition as an indicator of offspring metabolic syndrome risk in later life through epigenetic imprinting: a systematic review. Diabetes Metab Syndr. 11. 2017; 181(Suppl 2):S655–62. 10.1016/j.dsx.2017.04.021 28533070

[136] Berglind D , Müller P , Willmer M , Sinha I , Tynelius P , Näslund E , et al. Differential methylation in inflammation and type 2 diabetes genes in siblings born before and after maternal bariatric surgery. Obesity. 2016;24:250–61. 26637991 10.1002/oby.21340

[137] Van De Maele K , Gies I , Devlieger R . Effect of bariatric surgery before pregnancy on the vascular function in the offspring: protocol of a cross‐sectional follow‐up study. BMJ Paediatr Open. 2019;3:e000405. 10.1136/bmjpo-2018-000405 PMC6361362 30815589

[138] Price S , Nankervis A , Permezel M , Prendergast L , Sumithran P , Proietto J . Health consequences for mother and baby of substantial pre‐conception weight loss in obese women: study protocol for a randomized controlled trial. Trials. 2018;19:248. 29690917 10.1186/s13063-018-2615-6 PMC5926510

[139] Lloyd LJ , Langley‐Evans SC , McMullen S . Childhood obesity and risk of the adult metabolic syndrome: a systematic review. Int J Obes (Lond). 2012;36:1–11. 22041985 10.1038/ijo.2011.186 PMC3255098

[140] Lloyd LJ , Langley‐Evans SC , McMullen S . Childhood obesity and adult cardiovascular disease risk: a systematic review. Int J Obes (Lond). 2010;34:18–28. 19434067 10.1038/ijo.2009.61

[141] Arenz S , Rückerl R , Koletzko B , von Kries R . Breast‐feeding and childhood obesity‐a systematic review. Int J Obes Relat Metab Disord. 2004;28:1247–56. 15314625 10.1038/sj.ijo.0802758

[142] Horta BL , Loret de Mola C , Victora CG . Long‐term consequences of breastfeeding on cholesterol, obesity, systolic blood pressure and type 2 diabetes: a systematic review and meta‐analysis. Acta Paediatr. 2015;104:30–7. 26192560 10.1111/apa.13133

[143] Koletzko B , von Kries R , Closa R , Escribano J , Scaglioni S , Giovannini M , et al. Lower protein in infant formula is associated with lower weight up to age 2 y: a randomized clinical trial. Am J Clin Nutr. 2009;89:1836–45. 19386747 10.3945/ajcn.2008.27091

[144] Ballard O , Morrow AL . Human milk composition: nutrients and bioactive factors. Pediatr Clin North Am. 2013;60:49–74. 23178060 10.1016/j.pcl.2012.10.002 PMC3586783

[145] Bouret SG , Simerly RB . Minireview: Leptin and development of hypothalamic feeding circuits. Endocrinology. 2004;145:2621–6. 15044371 10.1210/en.2004-0231

[146] Warneke W , Klaus S , Fink H , Langley‐Evans SC , Voigt JP . The impact of cafeteria diet feeding on physiology and anxiety‐related behaviour in male and female Sprague‐Dawley rats of different ages. Pharmacol Biochem Behav. 2014;116:45–54. 24269545 10.1016/j.pbb.2013.11.016

[147] Leghi GE , Lai CT , Narayanan A , Netting MJ , Dymock M , Rea A , et al. Daily variation of macronutrient concentrations in mature human milk over 3 weeks. Sci Rep. 2021;11:10224. 33986316 10.1038/s41598-021-89460-5 PMC8119942

[148] Ward E , Yang N , Muhlhausler BS , Leghi GE , Netting MJ , Elmes MJ , et al. Acute changes to breast milk composition following consumption of high‐fat and high‐sugar meals. Matern Child Nutr. 2021;17:e13168. 33660402 10.1111/mcn.13168 PMC8189213

[149] Pearce J , Taylor MA , Langley‐Evans SC . Timing of the introduction of complementary feeding and risk of childhood obesity: a systematic review. Int J Obes (Lond). 2013;37:1295–306. 23736360 10.1038/ijo.2013.99

[150] Pearce J , Langley‐Evans SC . The types of food introduced during complementary feeding and risk of childhood obesity: a systematic review. Int J Obes (Lond). 2013;37:477–85. 23399778 10.1038/ijo.2013.8

[151] Pearce J , Langley‐Evans SC . Comparison of food and nutrient intake in infants aged 6‐12 months, following baby‐led or traditional weaning: a cross‐sectional study. J Hum Nutr Diet. 2022;35:310–24. 34476858 10.1111/jhn.12947

[152] Rowan H , Lee M , Brown A . Estimated energy and nutrient intakes for infants following baby‐led and traditional weaning approaches. J Hum Nutr Diet. 2022;35(2):325–36. 10.1111/jhn.12981 34927773 PMC9511768

[153] Langley‐Evans SC . Complementary feeding: should baby be leading the way? J Hum Nutr Diet. 2022;35:247–9. 35066946 10.1111/jhn.12988 PMC9303566

[154] Ma RCW , Popkin BM . Intergenerational diabetes and obesity ‐ a cycle to break? PLoS Med. 2017;14:e1002415. 29088227 10.1371/journal.pmed.1002415 PMC5663330

[155] Hieronimus B , Ensenauer R . Influence of maternal and paternal pre‐conception overweight/obesity on offspring outcomes and strategies for prevention. Eur J Clin Nutr. 2021;75:1735–44. 34131301 10.1038/s41430-021-00920-7 PMC8636250

[156] Su L , Patti ME . Paternal nongenetic intergenerational transmission of metabolic disease risk. Curr Diab Rep. 2019;19:38. 31127415 10.1007/s11892-019-1163-0

